# A study protocol of the effectiveness of PEGASUS: a multi-centred study comparing an intervention to promote shared decision making about breast reconstruction with treatment as usual

**DOI:** 10.1186/s12911-017-0543-0

**Published:** 2017-10-02

**Authors:** Diana Harcourt, Nicole Paraskeva, Paul White, Jane Powell, Alex Clarke

**Affiliations:** 1 Department of Health & Social Sciences, Centre for Appearance Research, Faculty of Health & Applied Sciences University of the West of England, Coldharbour Lane, Bristol, BS16 1QY UK; 2 Department of Engineering Design & Mathematics, Faculty of Environment & Technology, University of the West of England, Bristol, BS16 1QY UK; 3 Public Health and Well Being Research Group, Department of Health & Social Sciences, Faculty of Health & Applied Sciences University of the West of England, Bristol, BS16 1QY UK

**Keywords:** Pegasus, Breast reconstruction, Shared decision making, Breast cancer, Mastectomy, Risk reducing mastectomy, Ductal carcinoma in situ (DCIS)

## Abstract

**Background:**

Increasingly, women elect breast reconstruction after mastectomy. However, their expectations of surgery are often not met, and dissatisfaction with outcome and ongoing psychosocial concerns and distress are common. We developed a patient-centered intervention, PEGASUS:(Patients’ Expectations and Goals: Assisting Shared Understanding of Surgery) which supports shared decision making by helping women clarify their own, individual goals about reconstruction so that they can discuss these with their surgeon. Our acceptability/feasibility work has shown it is well received by patients and health professionals alike. We now need to establish whether PEGASUS improves patients’ experiences of breast reconstruction decision making and outcomes. The purpose of this study is, therefore, to examine the effectiveness of PEGASUS, an intervention designed to support shared decision making about breast reconstruction.

**Methods:**

A multi-centered sequential study will compare the impact of PEGASUS with usual care, in terms of patient reported outcomes (self-reported satisfaction with the outcome of surgery, involvement in decision making and in the consultation) and health economics. Initially we will collect data from our comparison (usual care) group (90 women) who will complete standardized measures (Breast-Q, EQ5D -5 L and ICECAP- A) at the time of decision making, 3, 6 and 12 months after surgery. Health professionals will then be trained to use PEGASUS, which will be delivered to the intervention group (another 90 women completing the same measures at the time of decision making, and 3, 6 and 12 months after surgery). Health professionals and a purposefully selected sample of participants will be interviewed about whether their expectations of reconstruction were met, and their experiences of PEGASUS (if appropriate).

**Discussion:**

PEGASUS may have the potential to provide health professionals with an easily accessible tool aiming to support shared decision making and improve patients’ satisfaction with breast reconstruction. Results of this study will be available at the end of 2019.

**Trial registration:**

ISRCTN 18000391 (DOI 10.1186/ISRCTN18000391) 27/01/2016.

**Electronic supplementary material:**

The online version of this article (10.1186/s12911-017-0543-0) contains supplementary material, which is available to authorized users.

## Background

Mastectomy can have a considerable, long-term psychosocial impact for women worldwide. In England alone, more than 15,000 women undergo mastectomy each year and over 5000 elect breast reconstruction (BR) to surgically recreate a breast shape [1]. Options regarding BR timing and type are numerous, requiring women to make complex decisions amidst the emotional upheaval associated with cancer. Although BR aims to improve quality of life and appearance concerns, a national audit [1] reported worrying levels of dissatisfaction with surgical outcome, supporting previous reports of ongoing pain [2] and scarring [3]. BR is not a panacea for the distress of mastectomy [4] - almost half patients report some regret [5].

Women seeking BR anticipate it will restore a sense of ‘normality’, but their expectations of outcomes are often unclear [6, 7]. The decision is ‘preference-sensitive’ [8] because the ‘right choice’ depends on individuals’ personal preferences rather than generic treatment factors. Clarifying each patient’s preferences and values is imperative, central to shared decision making (SDM) [9] and key to patient-centred care [10]. However, treatment decision making often triggers breakdowns in communication between patients and health professionals (HPs) [11]. Patients and health professionals differ when rating the importance of facts and goals guiding decisions about reconstruction [8]. Whilst women make choices by drawing comparisons with their pre-cancer appearance, health professionals draw on their clinical experience of a wider range of post-surgical outcomes including appearance, pain, impact on physical activities, body image and wellbeing.

Women dissatisfied with the outcome of BR may seek further, corrective surgery (with implications for healthcare resources and distress for patients), and may maintain avoidance behaviours (e.g. intimacy, clothing choices) that BR was intended to reduce [6]. We surmise that if women have realistic expectations, then patient dissatisfaction, well-being and requests for additional surgery would improve. Although HPs need to ask women about their priorities and concerns [8], promote realistic expectations [12, 13], and support them making high quality decisions by empowering them through SDM [14], there are no interventions to help them elicit patients’ values, expectations and preferences. Instead, support typically focuses on information provision (e.g. websites, leaflets). However, increasing the amount of information available does not address erroneous expectations since it reinforces patients as passive recipients rather than actively engaged in setting clear, patient-centred goals (an approach associated with positive experiences and outcomes [15]. SDM has been heralded as a means of improving patient reported outcomes and satisfaction with cancer care [16] (particularly concerning preference sensitive decisions [17]) but implementation is difficult and slow [18]. Some decision aids for mastectomy patients are available [19, 20] but these are not BR-specific or focused around a face-to-face discussion centred on patients’ individual needs and the practicalities and possibilities of surgery. Nor do they explicitly help HPs and patients with the challenge of implementing SDM in this difficult and emotive situation. Clinicians report concerns that online interventions such as BRESDEX.com cannot be tailored to patients’ individual needs, and could replace nurses’ roles and induce patient anxiety if not provided under clinical supervision [20]. Recently, attention has shifted to decision coaching to facilitate patients’ preparation for SDM about preference sensitive decisions [21].

We developed an intervention to facilitate SDM by specifically helping patients and HPs clarify each woman’s motivations for BR (PEGASUS: Patients’ Expectations and Goals: Assisting Shared Understanding of Surgery) which is designed to elicit each patient’s own expectations of what she wants BR to achieve, facilitate setting patient centred goals (what she considers a successful outcome), and aid discussion of both physical and psychosocial expectations, goals and outcomes with their surgical team. PEGASUS is delivered to women previously informed about their reconstructive options, since physique and health status may render some procedures inappropriate. Unlike a purely paper-based intervention [19], PEGASUS involves meeting a decision facilitator (a specialist nurse/psychologist trained in its use who is referred to as the PEGASUS Coach) during which the patient is helped to identify her individual BR goals, what would indicate a successful outcome and the importance of each goal. She takes the completed PEGASUS sheet into the surgical consultation where it is used to inform shared goals and promote concordance between the patient and surgeon, so they approach surgical outcomes as a shared endeavour [22].

Interventions encouraging patients to prepare for and actively engage in consultations effectively improve satisfaction and health outcomes [23, 24]. PEGASUS facilitates the disclosure and discussion of expectations, enabling the surgeon to decide the extent to which they are realistic and, if necessary, take appropriate steps to address unrealistic expectations (e.g. explain the likely outcomes further, show more photographs). PEGASUS is a novel, well-accepted intervention that helps women express what they want and assist health professionals ‘diagnose’ their patients’ preferences – failure to do this is common and warrants interventions to “transform the role of patients in the NHS from passive users into active and engaged partners in care” ([25] pviii). Our work follows recommendations for developing and evaluating complex interventions [26]. Feasibility and acceptability testing is complete (conducted under NRES reference 11/NW/0788) and showed that PEGASUS is acceptable to both patients and health professionals alike [27]. We now need to establish whether using PEGASUS offers benefits over usual care – this is the aim of the current study.

## Method

### The main research question

- Is a patient-centred, goal-focussed intervention (PEGASUS) that was developed to support shared decision making (SDM) for women contemplating breast reconstruction (BR) effective in terms of patient reported outcomes and costs?

### Aims of the study

- To assess the efficacy of an intervention designed to:
  - ◦ elicit patients’ expectations of what reconstructive breast surgery will achieve
  - ◦ facilitate setting of patient-centred goals
  - ◦ facilitate discussion of these expectations and goals with the patient’s surgical team.
- To explore whether the PEGASUS intervention improves patient reported outcomes (Breast Q) and reduces decisional conflict amongst women offered the option of breast reconstruction.
- To explore the economic costs of delivering the PEGASUS intervention compared with the costs of treatment as usual.
- To explore patients’ and health professionals’ experiences of using the PEGASUS intervention and its implementation within breast reconstruction services.

The research will be overseen and informed by the expertise of an advisory group consisting of patient representatives, a surgeon, clinical psychologist, specialist nurse, breast cancer charity representative and members of the research team.

### Design

A mixed methods (qualitative and quantitative methods), multi-centred, non-randomised between subjects, before-and-after design comparing usual care (control) with the intervention (PEGASUS). Randomised Controlled Trials (RCTs) are not necessarily suited for psychosocial research [28]. In this study it is inappropriate to randomize participants since contamination effects between conditions are likely if surgeons must focus some consultations around the patient goals offered by PEGASUS, but revert to usual care in others. In this non-randomised design, the control group comprises consenting patients receiving usual care. On completion of the control group recruitment, the health professionals receive PEGASUS training, prior to intervention group recruitment. Semi-structured interviews will be conducted with a selection of participants and health professionals involved in the PEGASUS intervention condition. Intervention consultations will be recorded (with permission) and assessed against a study-specific checklist to check the fidelity of the intervention.

### Trial design characteristics

**Fig. 1:**
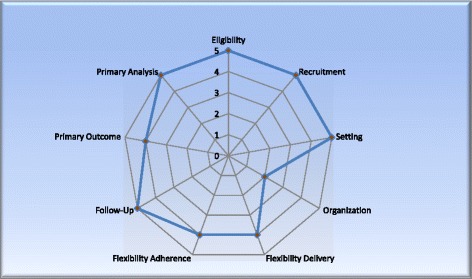
PRECIS-2

The characteristics of the PEGASUS trial were assessed using PRECIS-2 [29]. PRECIS-2 is a tool specifically designed to assess trial design on a continuum of pragmatic-explanatory across nine criteria: eligibility, recruitment, setting, organisation, flexibility delivery, flexibility adherence, follow-up, primary outcome and primary analysis. Pragmatic trials aim to explore whether an intervention works in real-life settings whereas exploratory trials aim to explore whether an intervention works under ideal conditions [29, 30]. Figure 1 illustrates the trial design based on the nine criteria. Eligibility, recruitment, setting, follow-up and primary analysis follow a pragmatic approach, whereas organisation, flexibility delivery, flexibility adherence and primary analysis fall along the pragmatic-explanatory continuum.

### Sites

Data collection will take place at hospital sites (Bristol, Bath, Truro, Winchester, and Cardiff) with comprehensive breast reconstruction services. The researcher will maintain weekly contact with each site, where clinic staff will identify eligible participants, provide study information and give the researcher the details of those who consent to participate.

### Sample size

One hundred eighty women over the age of 18 who have been diagnosed with breast cancer or DCIS (Ductal Carcinoma in Situ) or are seeking risk reducing surgery due to a strong family history of breast cancer, and who are considering breast reconstruction following mastectomy. We will record how many women are eligible for the study, how many participate and record reasons for refusal, where possible.

### Power calculations

The primary outcome measure is the Breast-Q (breast reconstruction) [31] with psychosocial wellbeing, sexual well-being, physical well-being and 5 satisfaction subscales applicable pre- and post-op and an expectations subscale applicable pre-operatively only. Sample size has been determined to ensure the simple comparison between the two groups 3–month post-operatively has at least 90% power (beta <= .20) using contemporary levels of significance (alpha = .05) in a two-sided test as this will ensure similar power requirements will be met for within group comparisons and for the anticipated interaction effect in the two-way ANOVA. An a priori power analysis based on a two-sided application of the independent samples t-test with an assumed lower estimate of a medium effect size (Cohen’s d = 0.5) indicates that at least 90% power would be achieved with a sample size of *n* = 90 per group. This stated power equates to at least 75% of women doing better in PEGASUS than they would have done with treatment as usual.

### Exclusion criteria

Women who, for any reason, are unsuitable for breast reconstruction or unable to participate in an intervention and study conducted in English will not be eligible to participate. We acknowledge this limitation, but do not currently have the resources needed to offer PEGASUS in other languages. If this study is successful we will explore its use with women with limited English proficiency (LEP) in future research.

### Intervention training

Specialist nurses, psychologists and surgeons at each site will be trained to use PEGASUS by AC (the clinical psychologist in the study team) and the study researcher (NP). Face-to-face training will be supplemented by a manual and video footage demonstrating its use, accessible throughout the study. Feedback on this training (elicited in the health professional interviews and questionnaires at the training sessions) will identify whether edits are needed prior to making training resources available through the PEGASUS website (www.pegasusdecisionmaking.com) in any subsequent roll-out of the intervention.

### Procedure (see Additional file 1)

Potential participants will be identified by clinic staff at each site from appropriate clinic lists.

At each participating site, the control group will be recruited before recruitment to the intervention condition begins. Staff will be trained in the use of the intervention once control group recruitment has been completed at their site.

Women identified as being eligible for the control (usual care) group will be given the study information sheet together with a consent form and questionnaire booklet and a stamped – return addressed envelope. Clinic staff will provide these materials and be on hand to address any questions from potential participants (and will contact the research team for assistance if there are any questions they are unable to answer).

Women identified as being eligible for the intervention group will be given the study information sheet, and the opportunity to ask questions about the study. Those interested in taking part will be given a consent form and an appointment at which they will complete the PEGASUS intervention - this appointment will be not less than 24 h after they have first been told about the study (exact details will depend on the specific study site).

During the intervention consultation, women who have chosen to take part in this study will have a discussion with the trained PEGASUS coach that will be aimed at clarifying their explicit goals and expectations about reconstructive surgery. Together, the coach and patient will complete the PEGASUS sheet – a single piece of paper which will summarise the conversation and list explicitly what the patient is hoping breast reconstruction will achieve. The participant will then take this PEGASUS sheet into their consultation with the breast reconstruction surgeon, who will have been trained to use the sheet, to encourage a discussion about the woman’s expectations, thereby framing the surgical discussion in terms of what it is that the patient is hoping to achieve.

We will seek consent from women in the intervention condition to record their PEGASUS consultations in order that we can assess the fidelity of the intervention.

We will ask all participants to complete the following self-report measures in the questionnaire booklet at baseline (i.e. pre-intervention for those in the PEGASUS condition, equivalent timing for those in the control condition) and 3, 6 and 12 months after surgery, together with visual analogue scales (VAS) to assess the extent to which expectations/goals have been met, and satisfaction with decision making and care. Measures will be available for completion in paper format and online. Statistical analysis will examine changes in the self-report scores over time.

### Measures

#### Primary outcome measure

Breast-Q (reconstruction or mastectomy versions, as appropriate) [31] is a widely used measure of quality of life specific to breast reconstruction.

#### Secondary outcome measures

EQ-5D-5 L (a measure of health-related quality of life) [32] and ICECAP-A [33] capabilities (what participants want to be able to do in various key aspects of life), shared decision making (Decisional Conflict scale [34], CollaboRATE [35] and visual analogue scales) and Decisional Regret (Decisional Regret Scale) [36].

Cost-utility analyses will be undertaken from a funder perspective over the timeframe of the study. Resources used at each stage of shared decision making will be recorded using a study-specific resource use checklist tool, which will be used with prices from published sources to estimate unit costs. Costs will be expressed in 2017 prices and uprated in line with inflation. Funder incremental costs (shared decision making with PEGASUS versus usual care) will be related to incremental change in EQ-5D-5 L scores to estimate (cost per Quality adjusted Life Year gained):

• Cost per Quality-adjusted Life Year gained based on the EQ5DL scores

• Average cost of implementing PEGASUS per site

• Average cost per patient

• Cost per hour of shared decision making

### Analysis

The analyses will be described in detail in a full Statistical Analysis Plan but, in brief, the design permits a robust statistical analysis using a linear mixed model with random intercepts for longitudinal data, a one-way ANCOVA with baseline pre-op measure as a covariate, and t-tests for prior reasoned group comparisons. An adjusted ANCOVA will be used to account for the between-subjects non-equivalent groups design and this will prevent an overestimation of treatment effect due to measurement error. Primary analyses will be performed under multiple imputation using Multiply Imputed Chained Equations (MICE) and repeated on a complete basis. Data validity checks and missing values analysis will be undertaken prior to descriptive and inferential analysis. PEGASUS is intended for use with any surgical group and we will compare delayed with immediate reconstruction patients for any systematic differences in outcome.

### Interviews

We will conduct a nested qualitative study that will involve semi-structured interviews with around 32 patients (8 per site; 4 intervention, 4 control) and 20 health professionals (PEGASUS Coaches and surgeons; 5 per site), exploring experiences of PEGASUS (where appropriate), patients’ expectations of reconstruction and if these were met. Patient interviews will be conducted around the 12 month follow up data collection, using Breast-Q scores (high, middle, low) to purposefully sample to ensure interviews are conducted with patients with varying outcomes. Telephone interviews will be conducted if preferred by the patient or health professional, or if face-to-face is not possible. Face-to-face interviews will be conducted at a time and place of the interviewee’s choosing (following standard University protocols for safe lone working). Interview recordings will be transcribed verbatim and subjected to thematic analysis [37]. Findings will be agreed by the research team, with feedback from the advisory group. Participants’ goals and expectations on the PEGASUS forms will be subjected to content analysis.

### Data storage/confidentiality

A data protection document describes the study’s safeguards for the storage and protection of confidential information, both held manually and on computers. This document is guided by the Caldicott principles for handling patient-identifiable information, The UK Data Protection Act (1998) [38] and Good Clinical Practice guidance [39]. All data will be treated as confidential. Only members of the research team who are also members of the breast reconstruction service at the participating sites will examine clinic and medical records in order to identify potential participants. Once consented, the research team based at the university may require access to participants’ medical records in order to collect and check health economic data. Permission to do this will be included on the participant consent form. Audio files (interviews and consultation recordings) and data will be stored on a password protected computer accessible only by the research team. Completed questionnaires and consent forms will be stored separately, in a locked filing cabinet.

### Research governance, safety and the conduct of the study

It was agreed that an independent data monitoring committee was not necessary and interim analyses will not be conducted. A PEGASUS steering group will be set up with an independent chair and members and will meet every 6 months. The steering group will provide advice and supervision of the study and will focus on the progress of the study, approaches to improve adherence, the collection of follow-up data, participant safety and consideration of new information. The management team (consisting of the principle investigator, clinical psychologist, researcher, health economist and statistician) will meet once-a-month to discuss study progress, protocol adherence and amendments for ethical approval. Access to the final dataset will be restricted to members of the study management group.

### Dissemination of research findings

Members of our steering and management groups will provide expert advice on the dissemination of the main outcomes to participants. The findings of this study will be disseminated via publications in peer-reviewed journals. Dissemination of the findings to health professionals and organisations supporting women with breast cancer will be planned and developed with stakeholders throughout the study.

### Study approvals

This study has been approved by the NRES Committee South Central - Berkshire B (reference 15/SC/0331) and the Faculty of Health and Applied Sciences Research Ethics Committee at the University of the West of England, Bristol. All necessary R&D approvals have been granted by the NHS study sites. The study has been accepted on the National Institute for Health Research (NIHR) Clinical Research Network (CRN) Portfolio.

**Research Sponsor:** University of the West of England, Bristol.

### Timeframe of the study

The trial started on the 1st of July 2015 and will be completed in June 2019.

## Discussion

This study will establish whether the PEGASUS intervention offers benefits over usual care for women offered the option of breast reconstructive surgery. Since it focuses on each patient’s individual, personal goals, PEGASUS offers a tool to support health professionals who are looking to provide patient-centred individualized care for women facing the choice of whether or not to undergo breast reconstruction of any sort and at any time (as an immediate or delayed procedure). It is not limited to any particular type or timing of reconstructive surgery since it is delivered after a patient has been given information by her surgical team about the particular procedures for which she is deemed to be a suitable candidate. We will collate information about the decisions women make about reconstructive surgery, as well as details of their diagnosis and other treatment in order to describe our sample.

This study does not employ randomization for the reasons outlined above. Instead, we are employing a rigorous design that is best suited to this specific clinical situation.

We plan future research to explore the acceptability and feasibility of the PEGASUS intervention with other patient groups, including adults seeking cosmetic surgical procedures and young people with craniofacial conditions.

## Additional file

Additional file 1: Flow chart of the PEGASUS trial. (DOCX 43 kb)

## Abbreviations

ANOVA: Analysis of variance
BR: Breast reconstruction
DCIS: Ductal carcinoma in situ
LEP: Limited English proficiency
MICE: Multiply imputed chained equations
NHS: National Health Service
PEGASUS: Patients’ Expectations and Goals: Assisting Shared Understanding of Surgery
RCT: Randomised controlled trials
SDM: Shared decision making
VAS: Visual analogue scales
