# Supplementary material for: A study protocol of the effectiveness of PEGASUS: a multi-centred study comparing an intervention to promote shared decision making about breast reconstruction with treatment as usual
Source: BMC Med Inform Decis Mak. 2017 Oct 2;17:143. doi: 10.1186/s12911-017-0543-0 (PMC5625613; doi:10.1186/s12911-017-0543-0)
Supplement: Additional file 1: — Flow chart of the PEGASUS trial. (DOCX 43 kb) [file 12911_2017_543_MOESM1_ESM.docx]

**Appendix 1: Flow chart of the PEGASUS trial**

Eligible participants identified from clinic lists and databases. Women who are offered and considering the option of immediate or delayed breast reconstruction (any type) because they have been diagnosed as having breast cancer or Ductal Carcinoma in Situ (DCIS), or are undergoing risk-reducing mastectomy are eligible to participate.

Eligible participants are given study information, consent form and baseline booklet to complete

Eligible: Women who are offered and considering the option of immediate or delayed breast reconstruction (any type) because they have been diagnosed as having breast cancer or Ductal Carcinoma in Situ (DCIS), or are undergoing risk-reducing mastectomy.

Eligible participants are given study information, consent form and baseline booklet to complete. There is a pre-paid envelope for return of consent form and baseline questionnaire to research team at UWE.

Collect participants contact details.

Research nurse approaches patient about the trial. If participant is interested, they are given information about the trial.

Participants wishing to take part in the trial complete informed consent (written).

All participants complete baseline questionnaire.

**PHASE 1**

90 women in the **control arm** (treatment as usual)

The training of participating sites in how to deliver the PEGASUS intervention.

.

**PHASE 2**

90 women in the **intervention arm** (take part in the PEGASUS intervention)

All participants are given the follow-up questionnaires at 3, 6 and 12 months post-surgery

Participants complete follow-up questionnaires

Qualitative Interviews

A purposeful sample of participants (control arm and intervention arm) and health professionals will be invited to participate in semi-structured interviews on their experiences.
